# *Dianthus superbus fructus *suppresses airway inflammation by downregulating of inducible nitric oxide synthase in an ovalbumin-induced murine model of asthma

**DOI:** 10.1186/1476-9255-9-41

**Published:** 2012-10-30

**Authors:** In-Sik Shin, Mee-Young Lee, Hyekyung Ha, Woo-Young Jeon, Chang-Seob Seo, Hyeun-Kyoo Shin

**Affiliations:** 1 Basic Herbal Medicine Research Group, Korea Institute of Oriental Medicine, 483 Expo-ro, Yusung-gu, Daejeon 305-811, Republic of Korea; 2 College of Veterinary Medicine, Chonnam National University, Gwangju, 500-757, Republic of Korea

**Keywords:** *Dianthus superbus fructus*, Asthma, Inflammation, Cytokine, Inducible nitric oxide synthase

## Abstract

**Background:**

*Dianthus superbus* has long been used as a herbal medicine in Asia and as an anti-inflammatory agent. In this study, we evaluated the anti-inflammatory effects of *Dianthus superbus fructus* ethanolic extract (DSE) on Th2-type cytokines, eosinophil infiltration, and other factors in an ovalbumin (OVA)-induced murine asthma model. To study the possible mechanism of the anti-inflammatory effect of DSE, we also evaluated the expression of inducible nitric oxide synthase (iNOS) in the respiratory tract.

**Methods:**

Mice were sensitized on days 0 and 14 by intraperitoneal injection of OVA. On days 21, 22 and 23 after initial sensitization, mice received an airway challenge with OVA for 1 h using an ultrasonic nebulizer. DSE was applied 1 h prior to OVA challenge. Mice were administered DSE orally at doses of 100 mg/kg or 200 mg/kg once daily from day 18 to 23. Bronchoalveolar lavage fluid (BALF) was collected 48 h after the final OVA challenge. Levels of interleukin (IL)-4, IL-13 and eotaxin in BALF were measured using enzyme-linked immunosorbent assays (ELISAs). Lung tissue sections were stained with hematoxylin and eosin for assessment of cell infiltration and mucus production with periodic acid shift staining, in conjunction with ELISA and western blot analyses for iNOS expression.

**Results:**

DSE significantly reduced the levels of IL-4, IL-13, eotaxin, and immunoglobulin (Ig) E, number of inflammatory cells in BALF, and inflammatory cell infiltration and mucus production in the respiratory tract. DSE also attenuated the overexpression of iNOS protein induced by OVA challenge.

**Conclusion:**

Our results suggest that DSE effectively protects against allergic airway inflammation by downregulating of iNOS expression and that DSE has potential as a therapeutic agent for allergic asthma.

## Background

The prevalence of allergic asthma has increased markedly in recent decades and has become an important problem [1]. Allergic asthma is caused mainly by allergens such as house dust, inhalants, foods, drugs, and animal dander and its symptoms include wheezing, coughing, tightness of the chest, and breathlessness [2-4]. These symptoms results from bronchoconstriction and bronchial mucosal thickening caused by eosinophilic airway inflammation, airway remodeling, and excessive mucus production. Allergic asthma is regarded as a chronic inflammatory disease of the airway characterized by airway inflammation and is mucus hypersecretion [5]. Airway inflammation in allergic asthma is under the control of complex regulatory mechanisms involving the releasing of T helper type 2 (Th2) cytokines, chemokines and other signaling molecules, including nitric oxide (NO) [5,6]. Under asthmatic condition, allergens processed by antigen-presenting cells induce the activation of Th2 cells, which release various cytokines, including interleukin (IL)-4, IL-5, and IL-13, which exacerbate the allergic response by increasing inflammatory cell infiltration into the airway and immunoglobulin (Ig)E secretion, and by stimulating mucus hypersecretion [7,8]. NO plays a crucial role in the pathogenesis of airway inflammation in allergic asthma. NO is synthesized from L-arginine by NO synthase (NOS), which exists in three isoforms: neuronal NOS (nNOS), endothelial NOS (eNOS), and inducible NOS (iNOS) [6]. Both nNOS and eNOS are considered constitutive, and are involved in vasodilation and bronchodilation [9]. iNOS seems to be involved mainly in immunomodulation [6]. iNOS is stimulated by many proinflammatory cytokines, and is responsible for prolonged production of higher concentration of NO [10]. iNOS production increases in asthmatic tissues and several types of inflammatory cells [11]. Recent studies have also shown that reducing iNOS expression attenuates the asthmatic response in the respiratory tract in various asthma models [10,12,13].

Currently, the standard medication for allergic asthma comprises combined therapy including inhaled corticosteroids, leukotriene receptor antagonists, and others [14]. However, these drugs produce side effects and do not treat many asthmatic individuals consistently [15]. There is a pressing need for new agents for treating allergic asthma, and herbal remedies are attracting increasing attention as potential agents for this condition. Many herbal remedies have shown protective effects in experimentally induced allergic asthma models [16].

*Dianthus superbus* has long been used as a herbal medicine in Asia and as an anti-inflammatory agent in the treatment of urinary infections, carbuncles, and carcinoma of the esophagus [17,18]. A previous study reported on the antioxidant and cytotoxic activities of *D. superbus fructus* ethanolic extract (DSE) [19]. However, the effects of DSE have not been reported in a murine model of allergic asthma. The aim of this study was to investigate the effects of DSE on airway inflammation in a murine model of ovalbumin (OVA)-induce allergic asthma.

## Methods

### Preparation of DSE

The dried fructus of *D. superbus* (300 g) were extracted three times by sonication for 1 h with 3 L of 70% ethanol. The extracted solution was filtered through filter paper and evaporated to dryness (56.89 g). The yield of ethanolic extract obtained was 18.96%.

### Animal

Specific pathogen-free female BALB/c mice (7 weeks old) were purchased from the Orient Co. (Seoul, Korea) and used after 1 week of quarantine and acclimatization. The mice were allowed sterilized tap water and standard rodent chow. All experimental procedures were approved by Korea Institute of Oriental Medicine Institutional Animal Care and Use Committee. This study was performed in compliance with the National Institutes of Health Guidelines for the care and use of laboratory animals and Korean national animal welfare law.

### OVA-induced allergic asthma

OVA sensitization and airway challenge were performed as described previously [5]. In brief, mice were sensitized on days 0 and 14 by intraperitoneal injection of 20 μg OVA emulsified in 2 mg aluminum hydroxide in 200 μL PBS buffer (pH 7.4). On days 21, 22 and 23 after initial sensitization, mice received an airway challenge with OVA (1%, w/v, in PBS) for 1 h using an ultrasonic nebulizer (NE-U12; Omron Corp., Tokyo, Japan). DSE was dissolved in PBS and was prepared fresh daily before each treatment. DSE was administered by gavage to mice at doses of 100 mg/kg or 200 mg/kg once daily from day 18 to 23. Negative and positive control mice were orally administered PBS or montelukast (30 mg/kg in PBS), respectively. Montelukast was developed as a cysteinyl leukotriene (cys-LT)-1 receptor antagonist [20] and was introduced into the market after successful clinical evaluation in patients with aspirin-sensitive asthma, nocturnal exacerbation of asthma, and allergic asthma [21].

Following OVA challenge, bronchoalveolar lavage fluid (BALF) samples were obtained from the mice and processed, and inflammatory cells were counted as described previously [5]. In brief, mice were sacrificed by intraperitoneal injection of pentobarbital (50 mg/kg; Hanlim Pharm. Co., Seoul, Korea) 48 h after the last challenge, and a tracheostomy was performed. To obtain BALF, ice-cold PBS (0.5 mL) was infused into the lung and withdrawn via tracheal cannulation three times (total volume 1.5 mL). Total inflammatory cell numbers were assessed by counting cells in at least five squares of a hemocytometer after exclusion of dead cells by Trypan blue staining. To determine differential cell counts, 100 μL of BALF was centrifuged onto slides using a Cytospin (Hanil Science Industrial, Seoul, Korea) (200 g, 4°C, 10 min). The slides were dried, and the cells were fixed and stained using Diff-Quik^®^ staining reagent (B4132-1A; IMEB Inc., Deerfield, IL), according to the manufacturer’s instructions. The supernatant obtained from BALF was stored at −70°C for biochemical analysis.

### Measurement of the levels of cytokines, chemokine, and IgE

The levels of IL-4, IL-13, and eotaxin in BALF were measured using enzyme-linked immunosorbent assay (ELISA) kits (BioSource International, Camarillo, CA) according to the manufacturer’s protocols. The levels of total IgE in BALF and plasma were measured using an ELISA. Microtiter plates were coated with anti-IgE antibodies (anti-mouse IgE; 10 g/mL; Serotec, Oxford, UK) in PBS-Tween 20, and incubated with BALF or plasma sample. The plates were then washed four times, and 200 μL of o-phenylenediamine dihydrochloride (Sigma-Aldrich, St. Louis, MO) was added to each well. The plates were incubated for 10 min in the dark and the absorbance was then measured at 450 nm.

### Immunoblotting

Equal amounts of total lung protein (30 μg) were heated at 100°C for 5 min, loaded onto 8% SDS-PAGE gels, and electrophoresed. The proteins were then transferred to a nitrocellulose membrane (at 100 V for 2 h). The membrane was blocked for 1 h with Tris-buffered saline containing 0.05% Tween-20 (TBST) plus 5% skim milk. It was incubated with anti-iNOS (1:1000 dilution; Santa Cruz Biotechnology, Santa Cruz, CA) and anti-β-actin (1:1000 dilution; Cell Signaling Technology, Danvers, MA) overnight at 4°C. The membrane was washed three times with TBST and then incubated with a horseradish peroxidase (HRP)-conjugated secondary antibody (1:3000 dilution; Jackson ImmunoResearch, West Grove, PA) for 1 h at room temperature. The membrane was again washed three times with TBST and developed using an enhanced chemiluminescence kit (ECL; Amersham Pharmacia Biotech, Uppsala, Sweden). For quantitative analysis, densitometric band values were determined using Chemi-Doc (Bio-Rad, Hercules, CA, USA).

### Histology

After BALF samples were obtained, lung tissue was fixed in 10% (v/v) neutral buffered formalin. Tissues were embedded in paraffin, sectioned at 4 μm thickness, and stained with H&E solution (hematoxylin, Sigma MHS-16, and eosin, Sigma HT110-1-32) and periodic acid−Schiff (PAS) (IMEB Inc., San Marcos, CA) to estimate inflammation and mucus production, respectively.

### Measurement of NO and prostaglandin E_2_ (PGE_2_) production in RAW 264.7 cells

The murine macrophage RAW 264.7 cell line was obtained from the American Type Culture Collection (ATCC, Rockville, MD, USA). The cells were cultured in Dulbecco’s modified Eagle’s medium (Gibco Inc., Grand Island, NY, USA) supplemented with 5.5% heat-inactivated fetal bovine serum (Gibco Inc.), penicillin (100 U/mL), and streptomycin (100 μg/mL) in a 5% CO_2_ incubator at 37°C. RAW 264.7 cells were plated at a density of 2.5 х 10^5^ cells/well in 48-well plates and incubated overnight. Cells were treated with lipopolysaccharide (LPS, 1 μg/mL) in the presence or absence of various concentrations of DSE. After incubation for 18 h, supernatants were analyzed for the levels of NO (Griess Reagent System, Promega Corp., Madison, WI, USA) and PGE_2_ (Cayman Chemical Co., Ann Arbor, MI, USA) according to the manufacturers’ protocols.

### Image capture and photomicrography

Photomicrographs were obtained using a Photometric Quantix digital camera running a Windows program, and montages were assembled in Adobe Photoshop 7.0. Images were croppedand corrected for brightness and contrast, but were not otherwise manipulated.

### Statistical analysis

Data are expressed as means ± standard error of the mean (SEM). Statistical significance was determined using analysis of variance (ANOVA) followed by a multiple comparison test with Bonferroni adjustment. P values < 0.05 or <0.01 were considered significant.

## Results

### Suppression of OVA-induced eosinophilia in BALF

**Figure 1:**
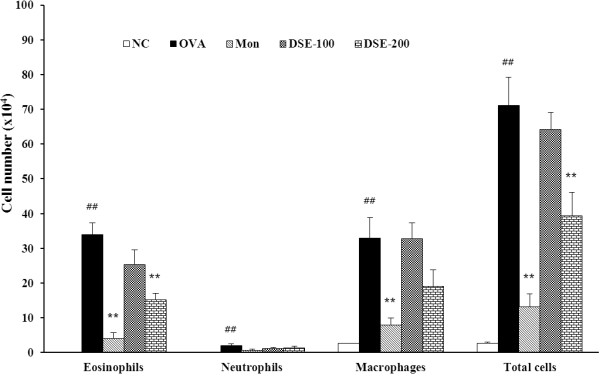
**DSE inhibits the recruitment of inflammatory cells in bronchoalveolar lavage fluid (BALF) of mice 48 h after the final OVA challenge. **Cells were isolated by centrifugation and stained with Diff-Quik^®^ stain reagent. Cell numbers were counted using a light microscope by counting cells in at least five squares of a hemocytometer after excluding dead cells using Trypan blue. NC, normal control mice treated with PBS only; OVA, OVA-sensitized/induced mice; Mon, montelukast (30 mg/kg) + OVA-sensitized/induced mice; DSE-100, DSE (100 mg/kg) + OVA-sensitized/induced mice; DSE-200, DSE (200 mg/kg) + OVA-sensitized/induced mice. Values are expressed as mean ± SEM (n = 6/group). ^##^Significantly different from NC, P < 0.01; ^**^significantly different from OVA, P < 0.01

OVA-induced mice had significantly more eosinophils, macrophages, neutrophils, and lymphocytes in BALF compared with the negative control mice (Figure 1). Montelukast-treated mice had significantly fewer inflammatory cells than OVA-induced mice. Similarly, DSE-treated mice had significantly fewer inflammatory cells, particularly eosinophils, compared with OVA-induced mice.

### Reduced levels of IL-4, IL-13, and eotaxin in BALF

**Figure 2:**
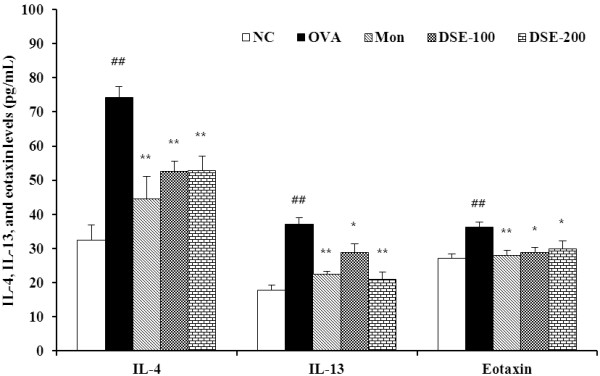
**DSE reduces the levels of IL-4, IL-5, and eotaxin in bronchoalveolar lavage fluid (BALF) of mice 48 h after the final OVA challenge. **OVA-induced mice had significantly higher levels of IL-4, IL-5, and eotaxin in BALF. DSE-treated mice had significantly lower levels of IL-4, IL-5, and eotaxin compared with OVA-induced mice. NC, normal control mice treated with PBS only; OVA, OVA-sensitized/induced mice; Mon, montelukast (30 mg/kg) + OVA-sensitized/induced mice; DSE-100, DSE (100 mg/kg) + OVA-sensitized/induced mice; DSE-200, DSE (200 mg/kg) + OVA-sensitized/induced mice. Values are expressed as mean ± SEM (n = 6/group). ^##^Significantly different from NC, P < 0.01; ^*,^^**^significantly different from OVA, P < 0.05 and < 0.01, respectively

The levels of IL-4 (74.3 ± 3.1 pg/mL, *P* < 0.01) and IL-13 (37.0 ± 1.9 pg/mL, *P* < 0.01) in BALF were significantly higher in OVA-induced mice compared with the negative controls. By contrast, montelukast-treated mice had significantly lower levels of IL-4 (44.4 ± 6.6 pg/mL, *P* < 0.01) and IL-13 (22.4 ± 0.9 pg/mL, *P* < 0.01) in BALF compared with OVA-induced mice. DSE-treated mice had significantly lower levels of IL-4 (52.6 ± 2.8 pg/mL in the 100 mg/kg DSE group, *P* < 0.01; 52.7 ± 4.2 pg/mL in the 200 mg/kg DSE group, *P* < 0.01) and IL-13 (28.9 ± 2.5 pg/mL in the 100 mg/kg DSE group, P < 0.05; 21.0 ± 2.1 pg/mL in the 200 mg/kg DSE group, *P* < 0.01) compared with OVA-induced mice. The level of eotaxin was also higher in OVA-induced mice (36.3 ± 1.5 pg/mL, *P* < 0.01) compared with the negative controls. Similar to the results for IL-4 and IL-5, eotaxin levels were lower in mice treated with montelukast (28.1 ± 1.4 pg/mL, *P* < 0.01) or DSE (28.8 ± 1.6 pg/mL in the 100 mg/kg DSE group, *P* < 0.01; 29.9 ± 2.3 pg/mL in the 200 mg/kg DSE group, *P* < 0.05) compared with the OVA-induced mice. These results are shown in Figure 2.

### Reduction in total IgE levels in BALF and plasma

As shown in Table 1, the level of total IgE in BALF was much higher in OVA-induced mice than in the negative controls, and was significantly lower in montelukast-treated mice compared with OVA-induced mice. The total IgE level in BALF was significantly lower in mice treated with DSE at 100 or 200 mg/kg compared with OVA-induced mice. As observed for IgE level in BALF, the level of total IgE in plasma was significantly lower in mice treated with 100 mg/kg DSE or with montelukast compared with OVA-induced mice. The total IgE level in plasma was nonsignificantly lower in 200 mg/kg DSE-treated mice compared with OVA-induced mice.

**Table 1:** The levels of total IgE in BALF and plasma

**Groups**	**BALF (pg/mL)**	**Plasma (ng/mL)**
NC	231.9 ± 96.99	0.4 ± 0.33
OVA	3117.5 ± 999.75^##^	17.6 ± 4.31^##^
Mon	1677.9 ± 513.30^**^	10.8 ± 3.30^*^
DSE-100	1651.9 ± 501.03^**^	10.0 ± 4.80^**^
DSE-200	2057.4 ± 465.84^*^	15.5 ± 3.43

NC; normal control mice treated with PBS only; OVA; OVA-sensitized/challenged mice; Mon; montelukast (30 mg/kg) + OVA-sensitized/challenged mice; DSE-100; DSE (100 mg/kg) + OVA-sensitized/challenged mice; DSE-200; DSE (200 mg/kg) + OVA-sensitized/challenged mice.

^##^Significantly different from NC, P < 0.01.

^*,^^**^significantly different from OVA, P < 0.05 and < 0.01, respectively.

### Attenuation of inflammatory cell infiltration and mucus production in lung tissue from OVA-induced mice

**Figure 3:**
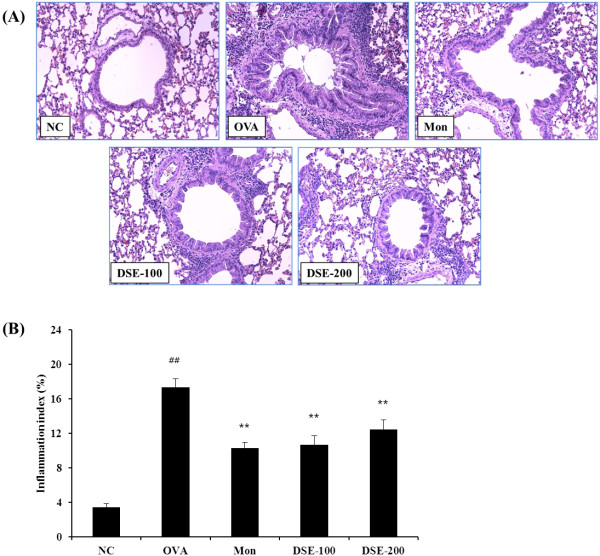
**DSE inhibits the recruitment of inflammatory cells to lung tissue of mice 48 h after the final OVA challenge. **(**A**) Histological examination of lung tissue with H&E stain (magnification x200). (**B**) Quantitative analysis of inflammatory cell infiltration in lung sections. NC, normal control mice treated PBS only; OVA, OVA-sensitized/induced mice; Mon, montelukast (30 mg/kg) + OVA-sensitized/induced mice; DSE-100, DSE (100 mg/kg) + OVA-sensitized/induced mice; DSE-200, DSE (200 mg/kg) + OVA-sensitized/induced mice. Values are expressed as mean ± SEM (n = 6/group). ^##^Significantly different from NC, P < 0.01; ^**^Significantly different from OVA, P < 0.01

We observed a marked infiltration of inflammatory cells into the peribronchiolar and perivascular lesions in lung tissues sections from OVA-induced mice compared with the negative control mice (Figure 3A). Most infiltrated inflammatory cells were eosinophils. By contrast, DSE-treated mice exhibited less infiltration of inflammatory cells into the periobronchiolar and perivascular lesions compared with the OVA-induced mice. Quantitative analysis of inflammation showed that DSE-treated mice had a significantly lower inflammation index compared with the OVA-induced mice (Figure 3B).

**Figure 4:**
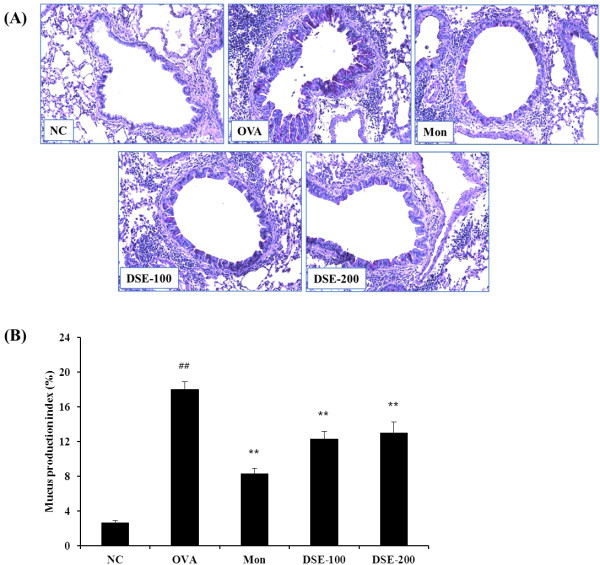
**DSE reduces mucus production in lung tissues of mice 48 h after the final OVA challenge. **(**A**) Histological examination of mucus secretion in lung tissue with periodic acid Schiff (PAS) (magnification x200). (**B**) Quantitative analysis of mucus production in lung sections. NC, normal control mice treated with PBS only; OVA, OVA-sensitized/induced mice; Mon, montelukast (30 mg/kg) + OVA-sensitized/induced mice; DSE-100, DSE (100 mg/kg) + OVA-sensitized/induced mice; DSE-200, DSE (200 mg/kg) + OVA-sensitized/induced mice. Values are expressed as mean ± SEM (n = 6/group). ^##^Significantly different from NC, P < 0.01; ^**^Significantly different from OVA, P < 0.01

Lung sections from OVA-induced mice stained with PAS exhibited overproduction of mucus and goblet cell hyperplasia (Figure 4A). Sections from montelukast-treated mice showed mild mucus production and goblet cell hyperplasia in the bronchial airway compared with OVA-induced mice. Quantitative analysis showed that DSE-treated mice had a significantly lower mucus production index compared with OVA-induced mice (Figure 4B).

### Reduction in iNOS expression in lung tissue from OVA-induced mice

**Figure 5:**
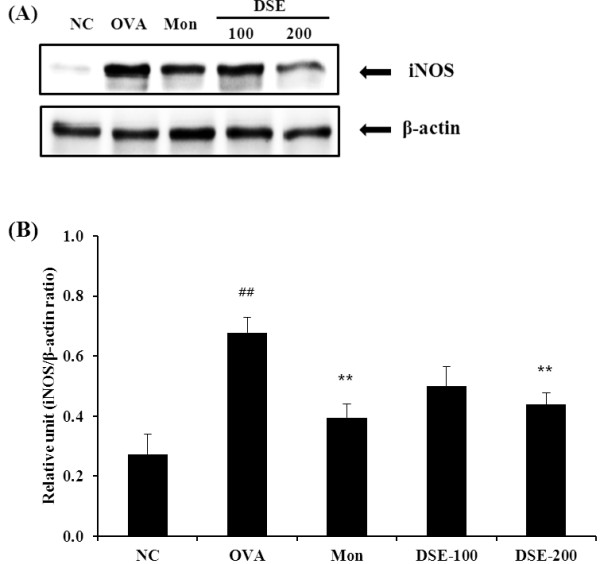
**DSE reduces the expression of iNOS protein in lung tissues of mice 48 h after the final OVA challenge. **(**A**) iNOS protein expression in the gel. (**B**) Relative units of iNOS protein expression. Relative units are expressed as the relative ratio of iNOS to β-actin. NC, normal control mice treated with PBS only; OVA, OVA-sensitized/induced mice; Mon, montelukast (30 mg/kg) + OVA-sensitized/induced mice; DSE-100, DSE (100 mg/kg) + OVA-sensitized/induced mice; DSE-200, DSE (200 mg/kg) + OVA-sensitized/induced mice. Values are expressed as mean ± SEM (n = 6/group). ^##^Significantly different from NC, P < 0.01; ^**^significantly different from OVA, P < 0.01

To identify the possible protective mechanism underlying the activity of DSE in airway inflammation, we investigated the expression of iNOS protein in relation to inflammation in lungs of OVA-induced mice. As shown in Figure 5A and B, OVA-induced mice showed significantly greater iNOS expression in the lung tissue compared with the negative controls. DSE-treated mice had significantly lower iNOS expression compared with OVA-induced mice.

### Suppression of NO and PGE_2_ production in RAW 264.7 cells

**Figure 6:**
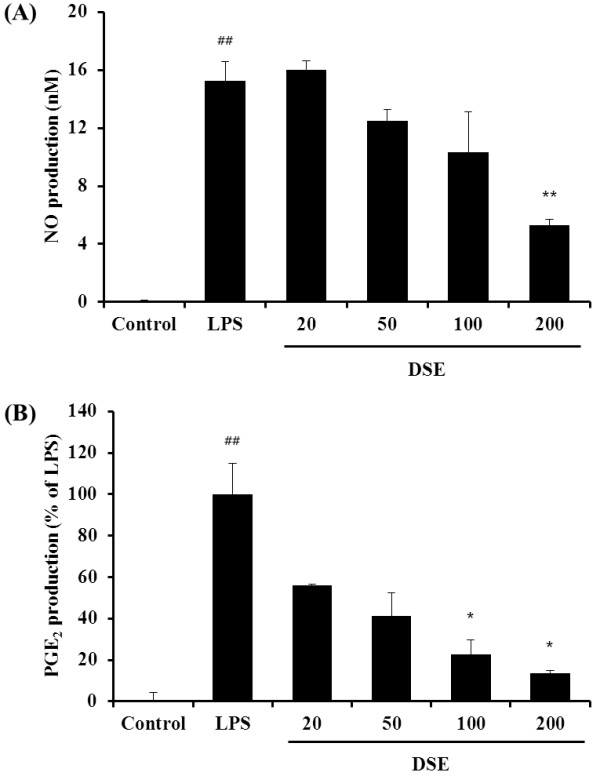
**DSE reduces the increased NO and PGE2 production induced by LPS stimulation in RAW 264.7 cells. **The production of NO (**A**) and PGE2 (**B**) was measured in culture medium of cells treated with DSE (20–200 ug/mL) and then co-stimulated with LPS for 18 h. Values are expressed as mean ± SEM. ^##^Significantly different from NC, P < 0.01; ^*^,^**^significantly different from OVA, P < 0.05 and < 0.01, respectively

We measured the cytotoxic effect of DSE on RAW 264.7 cells, exposed to various concentrations ranging from 20 to 200 μg/mL of the DSE for 24 h. Cell viability was then measured using the CCK-8 assay. Nontoxic concentrations were used for the subsequent experiments. LPS significantly increased NO production by RAW 264.7 cells (Figure 6A). By contrast, DSE significantly decreased NO production in dose dependent manner compared with only LPS-stimulated cells. In PGE_2_ production, DSE significantly reduced the increased PGE2 production induced by LPS stimulation, which was consistent with the results of NO production (Figure 6B).

## Discussion

In this study, we evaluated the anti-inflammatory effects of DSE in an OVA-induced murine asthma model and investigated its possible mechanism of action. OVA-induced mice showed eosinophilia, airway inflammation, mucus hypersecretion, and elevated levels of IL-4, IL-13 and eotaxin in BALF. OVA-induced mice had significantly greater expression of iNOS in the lung tissue. By contrast, DSE-treated mice had fewer inflammatory cells, in particular eosinophils, in BALF and lung tissue, and lower levels of IL-4, IL-13, and eotaxin in BALF compared with OVA-induced mice. Administration of DSE also reduced the expression of iNOS protein in lung tissue compared with OVA-induced mice. To our knowledge, this is the first study to show that DSE decreases airway inflammation in OVA-induced allergic asthma by reducing iNOS expression.

The pathogenesis of asthma is associated with increased infiltration of inflammatory cells and excessive mucus production into the airways [22]. Exposure to allergens induces the activation of Th2 cells, which release Th2 cytokines such IL-4, IL-5, and IL-13. Elevated Th2 cytokine levels stimulate infiltration of inflammatory cells into the respiratory tract [5]. In ours study, administration of DSE decreased both the numbers of inflammatory cells in BALF and the production of Th2 cytokines. These results were consistent with the results of the histopathological examination. OVA-induced mice showed severe inflammation in peribronchial lesions, whereas DSE-treated mice showed milder inflammation compared with OVA-induced mice. These results indicate that DSE effectively protected the mice from OVA-induced airway inflammation. Administration of DSE also decreased the levels of IgE in BALF and plasma and attenuated mucus hypersecretion into the respiratory tract. Previous studies have shown that Th2 cytokines increase IgE switching in B cells and IgE binding to FcεRI on mast cells and basophils, which accelerate the allergic response including the production of proinflammatory mediators and mucus secretion [5,7]. The results of our histopathological study are consistent with these findings. Our results suggest that DSE attenuates allergic response, including airway inflammation and mucus hypersecretion, by reducing Th2 cytokine release.

iNOS plays an important role in airway disease including allergic asthma [11]. Airway inflammation is attributed to increased expression and activity of iNOS, which increase NO production and consequently the numbers of eosinophils in the airway and airway inflammation [10,23]. Recent studies have shown that iNOS expression is closely related to Th2 cytokines release in attenuating the asthmatic response including airway constriction, inflammatory cell infiltration, and remodeling processes [9,24,25]. In this study, the expression of iNOS was much higher in OVA-induced mice compared with the negative control mice and DSE significantly attenuated the overexpression of iNOS induced by OVA in lung tissue. These results were consistent with the changes in Th2 cytokine production and histopathological examination of lung tissue. In addition, DSE significantly reduced the increased NO production induced by LPS stimulation in RAW 264.7 cells, which is consistent with the results of western blot. Taken together, these results suggest that the reduction in iNOS expression is one possible mechanism to which DSE attenuates airway inflammation in experimentally induced allergic asthma.

## Conclusion

DSE is used traditionally for treatment of inflammatory diseases in Korea and China. However, only limited information is available about pharmacological effects of DSE. In particular, no data are available on the protective effect of DSE in allergic asthma. DSE was recently reported to suppress IgE production in a human B cell line and a murine model of peanut allergy [26]. Our results show that DSE attenuates OVA-induced airway inflammation in a murine model of asthma by downregulation of iNOS expression. These findings suggest that DSE may be useful as an adjuvant therapy for asthmatic patients.

## Competing interest

The authors declare that they have no competing interests.

## Authors’ contributions

ISS, MYL and HKS participated in the design of the study data analyses and manuscript preparation. HSL, WYJ and HH conducted the assays and analyses. CSS provided DSE samples. All authors read and approved the final manuscript.
